# Nurse Leaders’ Experiences of Implementing Career Advancement Programs for Nurses in Iran

**DOI:** 10.5539/gjhs.v7n5p73

**Published:** 2015-02-24

**Authors:** Mohammad Reza Sheikhi, Masoud Fallahi Khoshknab, Farahnaz Mohammadi, Fatemeh Oskouie

**Affiliations:** 1 Department of Nursing, University of Social Welfare & Rehabilitation Sciences,(USWR) Tehran, Iran; 2 Centers for Nursing Care Research and School of Nursing and Midwifery, Iran University of Medical Sciences, Tehran, Iran

**Keywords:** nursing, career mobility, qualitative research, professional development

## Abstract

**Background and purpose:**

Career advancement programs are currently implemented in many countries. In Iran, the first career advancement program was Nurses’ Career Advancement Pathway. The purpose of this study was to explore nurse leaders’ experiences about implementing the Nurses’ Career Advancement Pathway program in Iran.

**Methods:**

This exploratory qualitative study was conducted in 2013. Sixteen nurse managers were recruited from the teaching hospitals affiliated to Shahid Behesthi, Qazvin, and Iran Universities of Medical Sciences in Iran. Participants were recruited using purposive sampling method. Study data were collected through in-depth semi-structured interviews. The conventional content analysis approach was used for data analysis.

**Results:**

participants’ experiences about implementing the Nurses’ Career Advancement Pathway fell into three main categories including: a) the shortcomings of performance evaluation, b) greater emphasis on point accumulation, c) the advancement-latitude mismatch.

**Conclusion:**

The Nurses’ Career Advancement pathway has several shortcomings regarding both its content and its implementation. Therefore, it is recommended to revise the program.

## 1. Introduction

Career advancement (CA) and staff ranking programs for nurses have been developed and implemented for more than four decades (Fusilero et al., 2008; Pierson, Liggett, & Moore, 2010) and are commonly referred to as ‘clinical ladder’ or career ladder programs (Sonmez & Yildirim, 2009). CA programs specifically for nurses were initially developed in 1972 by Marie Zimmer (Fusilero et al., 2008; Riley et al., 2009), which was originated from Benner’s Novice to Expert theory (Korman & Eliades, 2010). In this program, nurses advance to higher positions through developing and demonstrating professional competencies and abilities (Fusilero et al., 2008; Pierson et al., 2010).

In Iran, the first CA program was the Nurses’ Career Advancement Pathway (NCAP), which was developed and implemented by the Ministry of Health and Medical Education in 2003. The aim of the NCAP was to improve nurses’ motivation and productivity. In this program, nurses are usually evaluated and ranked based on performance evaluation procedures, self-evaluations, participation in continuing education programs (CEPs), and work experience (Nekoei Moghadam, Arabpour, Majidi, & Molaei, 2010). The NCAP ranks nurses in four levels including basic, senior, expert, and excellent; each level lasts for six years. In order to rise to a higher level, nurses need to accumulate the necessary points through actively participating in in-service education programs and demonstrating professional competencies and abilities. Advancement to higher level is associated with 5–10% increase in the annual salary. On the other hand, the NCAP is a powerful tool for nurse managers that help them facilitate nurses’ CA. Indeed, it is a standard system for improving nurses’ capabilities, particularly junior nurses and provides opportunities for effective learning and professional development (Nekoei Moghadam et al., 2010; Rezapour, 2005).

Evidence abounds that achieving career advancement program in nursing leads to nurses’ performance improvement. Several reports indicated that implementation of advancement programs resulted in retention of experienced nurses, decrease nursing staffs ’attrition and turnover, strengthen their motivation, and enhance the quality of nursing care (Fusilero et al., 2008; Korman & Eliades, 2010). Numerous reports demonstrated that well-educated nurse who participate in ongoing career advancement program are invaluable to achieving nursing goals for improving the quality of patient care, clinical skills and professional growth of new nurses, increasing staff productivity (Rahimaghaee, Nayeri, & Mohammadi, 2010; Riley et al., 2009).

Despite nearly a decade from the introduction of the NCAP, the aims of this program have not yet been fulfilled. The results of a quantitative study revealed that the NCAP did not improve nurses’ performance (Nekoei Moghadam et al., 2010). This information would be helpful to inform nurse managers as they evaluate and implement career advancement for nurse in NCAP in their organization. On the other hand, nurse managers are an important part of healthcare system, which are considered as integration between strategic goals and realities in the healthcare system. Thus, their experiences can be used by senior managers for developing and implementing strategic plans for improving the effectiveness of healthcare organization and the quality of care. Exploring their experiences about the CA programs can provide valuable information about the strengths and weaknesses of CA programs (Adeniran, Bhattacharya, & Adeniran, 2012; Adeniran, Smith-Glasgow, Bhattacharya, & Xu, 2013). In addition, identifying and removing barriers to the effective implementation of CA programs can facilitate the fulfillment of the strategic aims of healthcare systems and improve nurses’ motivation for participating in these programs (Havill, 2010). To the best of our knowledge, few studies have been conducted on evaluating the NCAP and bridge this gap. Therefore, this study was conducted to explore nurse managers’ experiences during implementing NCAP.

## 2. Methods

This study was an exploratory qualitative design, which allows deep exploration of the lives of nurses, as experienced and voiced by them. Qualitative method includes a systematic and organized interpretation of textual data that are gathered from context through conducting interviews and observations (Elo & Kyngäs, 2008). Accordingly, it is an effective method for exploring nurse managers’ experiences about the NCA. In this study, sixteen nurse manager including head-nurses, supervisors, hospital nurse managers, and university nurse managers participated; they were selected using purposive sampling. The investigators strived to recruit a maximum variation sample to include diverse experiences in the study. Data collection continued until reaching data saturation.

The study setting was hospitals affiliated to Shahid-Behesthi, Qazvin, and Iran Universities of Medical Sciences, Iran. Data were gathered using face-to-face semi-structured interview method. The research questions were formulated based on the aim of this study. Then, as researchers we particularly focused on the process of implementing the NCAP. Probing questions were also employed to enrich the data. All interviews were conducted by the first author in a silent room located in the participants’ workplace. Interviews lasted between 45 to 100 minutes with a mean of 60 minutes. Interviews were recorded using a sound recorder and immediately transcribed them verbatim. The anonymity of the study participants was maintained by allocating a number to each interview. We employed the conventional qualitative content analysis approach for data analysis. The main aim of conventional qualitative content analysis is to condense raw data and provide a detailed description of them. Data were analyzed as follows:

a) Transcribing the interviews, reading through transcriptions several times as to comprehend the material in its entirety
b) Dividing the text into meaningful units, ensuring “dense” and rich narrative
c) Abstracting the condensed units of meaning and labeling them with ascribed codes
d) Sorting the codes into sub-themes based on comparisons regarding their similarities and differences
e) Formulating themes as the expression of the latent content of the text.

We maintained the trustworthiness of the study findings by using the Guba and Lincoln’s credibility: confirm ability, dependability, and transferability criteria (Streubert Speziale & Carpenter, 2003). Accordingly, techniques such as member- and peer-checking and constant comparison were used for enhancing the credibility of the findings. The dependability of the findings was also established by employing the external debriefing, analyzer triangulation, and member-checking techniques. Accordingly, we provided the study participants a summary of the generated codes and interview transcripts and asked them to determine how much the analyses reflected their CA experiences. We also invited three nursing faculties to assess and confirm the process of data analysis for enhancing the confirm ability of the findings. Finally, we provided thick descriptions of the study participants’ experiences for increasing the transferability of the findings.

All participants were informed about the voluntary nature of participation, with the option to withdraw from the study at any time. A written informed consent was obtained from all participants. We also guaranteed the confidentiality of the participants’ personal information; they were ensured that the study findings would be reported and published anonymously.

## 3. Results

Totally, ten female and six male nurse managers with a mean age of 44 ±4.9 years participated in the study. The study participants’ experiences about implementing the NCAP fell into three themes including: shortcomings of performance evaluation, greater emphasis on point accumulation, and the advancement-latitude mismatch. Table (1) shows these themes with their corresponding subthemes.

**Table 1:** Themes and subthemes

Themes	Subthemes
Shortcomings of performance evaluation	Discontinuous evaluations
	incongruence between evaluation criteria and nurses’ job descriptions
	Subjective evaluations
Greater emphasis on point accumulation	Inattention to the effectiveness of CEPs
	Nurses’ higher motivation for participating in programs that have higher points
Advancement-latitude mismatch	Pay raise instead of role expansion
	Higher position without greater latitude
	Perceiving stagnancy following CA

### 3.1 Shortcomings of Performance Evaluation

According to our participants, CA-related performance evaluations were not performed effectively. This theme consisted of three subthemes including: discontinuous evaluations, incongruence between evaluation criteria and nurses’ job descriptions, and subjective evaluations. These categories are explained below.

#### 3.1.1 Discontinuous Evaluations

One of the main shortcomings of the current system of performance evaluation was the long interval between evaluations, which negatively affected participants’ judgment about nurses’ performance. The participants highlighted a wide interval between evaluations, which interferes with making accurate judgments about nurses’ performance.

“*We probably can’t perform careful performance evaluations and make accurate judgments by such annual evaluations.”* (Hospital nurse manager with 15 years of nursing experience).

Most of the participants considered annual evaluations as problematic. They recommended continuous monthly evaluation for overcoming the disadvantages of annual ones.

“*We can minimize the challenges of annual evaluations by performing monthly or quarterly evaluations*.” (Hospital nurse manager with 22 years of nursing experience).

According to another participant: “*we had a logbook in which we write nurses’ strengths and weaknesses It help us to make more accurate judgment, but those logbooks have not be used anymore*”(participant 7, a supervisor with 18 years’ experience).

#### 3.1.2 Incongruence between Evaluation Criteria and Nurses’ Job Descriptions

Our participating nurse managers highlighted that the current performance evaluation programs pay little attention to nurses’ specialized services and instead focus greatly on their routine administrative duties.

“*The tool that we use for evaluating nurses is also used for evaluating other workers out of healthcare settings*.” (Hospital nurse manager with 22 years of nursing experience).

Moreover, the existing evaluation forms were not useful for effectively evaluating nurses’ communication skills, clinical competencies, and intellectual abilities.

“*We can’t evaluate many of nurses’ roles and tasks such as their clinical and communication skills*.” (Head nurse with 14 years of nursing experience).

#### 3.1.3 Subjective Evaluations

The third main shortcoming of performance evaluation was the subjective nature of the evaluations. Our participants directly experienced that senior managers evaluated them and staff nurses subjectively.

“*I have seen that evaluators firstly consider a total score for the intended nurse and then score the items of the evaluation form proportionately to that total score*.” (Hospital nurse manager with 22 years of nursing experience).

Some participants also highlighted that the items of the evaluation forms were occasionally unclear and non-specific. Accordingly, different evaluators might have had different understanding of such items.

“*Some of the items are scored qualitatively and hence an evaluator may judge about and score them subjectively*.” (Nurse leader with 19 years of nursing experience)

### 3.2 Greater Emphasis on Point Accumulation

The second main theme of the study was the higher importance of point accumulation. In the NCAP, nurses who are going to advance to the basic, senior, expert, and excellent levels need to earn at least 60, 70, 80, and 90 points, respectively. Moreover, they need to have a 300-, 250-, 200-, and 150-hour history of participating in in-service CEPs to become eligible for getting advanced to the basic, senior, expert, and excellent levels, respectively. Accordingly, one of the main incentives for nurses to participate in such programs was to accumulate the necessary points for CA. This category consisted of two sub-themes including inattention to the effectiveness of CEPs and nurses’ higher motivation for participating in programs that have higher points.

#### 3.2.1 Inattention to the Effectiveness of CEPs

The most important criterion for earning CEP-related points was merely earning the program certification. Accordingly, nurses usually pay little attention to the congruence between their own educational needs and the contents of the programs.

“*Nurses participate in programs that are irrelevant to their work. They just need program points and certificates*.” (Nurse leader with 18 years of nursing experience) Some participants also noted that nurses usually participated passively in CEPs. “*Nurses usually enroll in educational programs; however, they don’t participate in the programs actively and regularly*.” (Nurse leader with 12 years of nursing experience)

#### 3.2.2 Nurses’ Higher Motivation for Participating in Programs that Have Higher Points

According to our participants, nurses usually had higher motivation for participating in programs that have had higher points.

“*When we offer an educational course, all nurses participate in it; however, their main motivation for participation probably is not fulfilling their educational needs. Rather, they attend the course to earn its points*.” (Head nurse with 15 years of nursing experience).

Moreover, the participation rates in CEPs that had greater points/credits were higher.

“*When we initiate a program that has great points, all nurses, even those who are off-duty, participate in it*.” (Head nurse with 18 years of nursing experience).

### 3.3 The Advancement-Latitude Mismatch

The third main theme of the study was nurses’ advancement to higher positions without enjoying proportionate latitude. Although the main objective of CA is to promote nurses’ professional development, our participants highlighted that nurses’ CA was not associated with significant changes in the performances, roles, and job descriptions. Advancement-latitude mismatch was the greatest challenge of the NCAP. The three subthemes of this category were pay raise instead of role expansion, higher position without greater latitude, and perceiving stagnancy following CA

#### 3.3.1 Pay Rise Instead of Role Expansion

The key aim of the NCAP is to improve nurses’ performance, motivation, and productivity as well as the quality of care. However, the study participants noted that nurses’ advancement to higher positions was mostly associated with pay raise rather than role expansion and professional development.

“*Through implementing the Career Advancement Trajectory, we just increased nurses’ salaries and extended their financial benefits. However, the effectiveness of career advancement in improving nurses’ performance is not clear*.” (Hospital nurse manager with 22 years of nursing experience).

#### 3.3.2 Higher Position without Greater Latitude

In the NCAP, nurses advance to higher levels without experiencing any considerable changes in their job descriptions. Accordingly, they, in their new positions, are required to perform the same tasks that they used to perform in their previous positions in their new positions. Study participants highlighted that the job descriptions of the basic, senior, and expert nurses are the same as the job descriptions of senior, expert, and excellent nurses, respectively. They believed that CA programs would be successful only when they give nurses greater latitude and autonomy alongside with advancement to higher positions.

“*Basic nurses perform same tasks as excellent ones.”* (Head nurse with 16 years of nursing experience).

*“There is no significant difference among nurses who are in different levels*.” (Nurse leader with 15 years of nursing experience)

#### 3.3.3 Perceiving Stagnancy Following CA

Our participants highlighted that nurses usually did not feel being advanced to higher positions and being professionally developed due to lack of role expansion.

“*Most nurses recognize career advancement by its subsequent pay raise. They rarely have a sense of being really advanced to higher positions*.” (University nurse manager with 21 years of nursing experience).

“*Most of the nurses who advance to higher levels do not feel professionally developed*.” (University nurse manager with 19 years of nursing experience).

## 4. Discussion

This study was conducted to explore Iranian nurse managers’ experiences about the NCAP. Participants’ experiences of the NCAP fell into three themes including the shortcomings of performance evaluation, greater emphasis on point accumulation, and the advancement-latitude mismatch.

The first theme indicated that the implementation of performance appraisal in NCAP confronts difficulties such as discontinuous evaluation, incongruence between performance evaluation and nurses’ job description, subjective evaluation. Nurses’ managers described one of the shortcomings was discontinuous evaluations which were performed annually. Such wide-interval evaluations can negatively affect nurse managers’ judgments about nurses’ performance. The result of qualitative study revealed that nurses’ manager and nurses need regular meeting more than once a year to evaluate their development and the performance obstacles (Nikpeyma, Abed-Saeedi, Azargashb, & Alavi Majd, 2014). Currently, techniques such as direct observation of nurses’ performance, performing continuous short-interval evaluations, and using valid checklists for objective performance evaluation are recommended for eliminating the adverse effects of the intervening factors on CA. Clarck (2009) noted that systematic and continuous evaluation is vital to effective performance evaluation.

Another shortcoming of the NCAP was the incongruence between performance evaluation criteria and nurses’ job descriptions. Study participants reported that current performance evaluations are performed subjectively and by using general evaluation forms which mainly focus on nurses’ administrative and non-specialized tasks and neglect nurses’ professional skills and competencies. Lack of objective evaluation tools interferes with judging nurses’ performance by standards. Advancement system requires evaluative tools that capture essential behaviors and outcomes reflective of key nursing function. Marquis and Huston (2012) Found that using subjective evaluation tools as a major barrier to effective evaluation of nurses’ performance.

Participant declared that the lack of objectivity in evaluation tools greatly affects the appraisal process; it is an inaccurately rank performance of nurses. Some investigators hold one of the criticisms of nursing performance appraisal system is the general subjectivity of appraisal tools (Pazargadi, Afzali, Javadzadeh, & Alavi Majd, 2005).

The second theme included greater emphasis on point accumulation and certification. This way of earning points may result in nurses’ passive participation in CEPs without considering the congruence between their inherent educational needs and the contents of programs. Moreover, our participants highlighted that nurses are more eager for participating in CEPs that offer higher points. Similarly, Watts (2010) found that the main motivation for participating in national CEPs is earning the required certifications for CA.

The third main theme of the study was advancement-latitude mismatch, which entailed nurses’ advancement to higher positions without proportionate performance latitude. The only outcome of CA in the NCAP was pay increases. Higher satisfaction scores for clinical advancement may be indicative of the positive outcome for reward and recognition of the nurse (Korman & Eliades, 2010). Also evidence showed that the Iranian NCAP has been ineffective in improving nurses’ performance and expanding their roles after CA. Therefore, the findings suggested that modification need to be made in the advancement process (Nekoei Moghadam et al., 2010).

Many parties—including administrative managers and hospital administrators—contribute to the development and implementation of the NCAP. However, the study sample consisted only of nurse managers. Further studies are needed for exploring other aspects of the NCAP and other parties’ experiences about implementing the NCAP.

## 5. Conclusion

Study findings indicate that the NCAP has several shortcomings. Accordingly, it is recommended to revise nursing career advancement program. Evaluation criteria should be developed more objectively and more specifically on the basis of on nurses’ job descriptions and specialized tasks. Moreover, continuous evaluations, which are performed in short intervals, are recommended. In addition, CEPs should be developed and implemented to meet nurses’ real educational needs and improve the quality of care. Finally, the NCAP should be revised in such a way that gives nurses greater latitude and roles
